# Association of impaired fasting glucose and Type 2 Diabetes Mellitus with brain volume changes in Alzheimer’s Disease patients analyzed by MRI: a retrospective study

**DOI:** 10.7717/peerj.9801

**Published:** 2020-08-27

**Authors:** Weiwei Wang, Leongtim Wong, Lin Shi, Yishan Luo, Zhanhua Liang, Chunbo Dong, Qingwei Song, Tieli Liu, Qing Zhang, Ailian Liu, Yanwei Miao, Jianlin Wu

**Affiliations:** 1 Tianjin Medical University, Tianjin, China; 2 Radiology Department, The First Affiliated Hospital of Dalian Medical University, Dalian, Liaoning Province, China; 3 BrainNow Research Institute, Shenzhen, Guangdong Province, China; 4 Department of Imaging and Interventional Radiology, The Chinese University of Hong Kong, Hong Kong, China; 5 Neurology Department, The First Affiliated Hospital of Dalian Medical University, Dalian, Liaoning Province, China; 6 Radiology Department, Affiliated Zhongshan Hospital of Dalian University, Dalian, Liaoning Province, China

**Keywords:** Alzheimer’s disease, Impaired fasting glucose, Type 2 diabetes mellitus, Brain volume, Structural MRI, Brain atrophy, Pons, Fasting glucose level, Hyperglycemia

## Abstract

**Objectives:**

Alzheimer’s disease (AD), impaired fasting glucose (IFG), and Type 2 diabetes mellitus (T2DM) were reported associated with smaller brain volumes. Nevertheless, the association of hyperglycemia with brain volume changes in AD patients remains unclear. To investigate this issue, structural magnetic resonance imaging was used to compare brain volumes among AD patients with different fasting glucose levels.

**Methods:**

Eighty-five AD patients were divided into three groups based on their fasting glucose level as suggested by the American Diabetes Association: normal fasting glucose group (AD_NFG, *n* = 45), AD_IFG group (*n* = 15), and AD_T2DM group (*n* = 25). Sagittal 3D T1-weighted images were obtained to calculate the brain volume. Brain parenchyma and 33 brain structures were automatically segmented. Each regional volume was analyzed among groups. For regions with statistical significance, partial correlation analysis was used to evaluate their relationships with fasting glucose level, corrected for Mini-Mental State Examination score, age, education level, cholesterol, triglyceride, and blood pressure.

**Results:**

Compared with the AD_IFG and AD_NFG groups, the volume of pons in AD_T2DM group was significantly smaller. Fasting glucose was negatively correlated with pontine volume.

**Conclusions:**

T2DM may exacerbate pontine atrophy in AD patients, and fasting glucose level is associated with pontine volume.

## Introduction

Alzheimer’s disease (AD) is a progressive neurodegenerative disorder reportedly caused by the abnormal deposition of amyloid β and tau protein. AD is the main cause of dementia (Lane, Hardy & Schott, 2018). Type 2 diabetes mellitus (T2DM) is a phenotype of glucose metabolic disorder caused by insulin resistance (Handelsman et al., 2015). In the past, T2DM was considered as a systemic disease that induces complications in multiple peripheral organs, such as diabetic nephropathy and diabetic retinopathy. Recent studies have indicated that T2DM is closely related to the central nervous system impairment (Degen et al., 2016). AD and T2DM are both chronic non-communicable diseases that are highly prevalent and commonly diagnosed (Lane, Hardy & Schott, 2018; Ogurtsova et al., 2017).

Several previous studies have reported close connections between these two diseases (Greene et al., 2015; Lin, 2008; Rani et al., 2016). T2DM has been strongly associated with an increased risk of developing all types of dementia, including AD (Kadohara, Sato & Kawakami, 2017). Compared with healthy individuals, the incidence of AD in diabetic population is significantly higher (Zhang et al., 2017). Furthermore, prediabetes is an intermediate state between normoglycemia and diabetes, and is also considered to be a risk factor for AD development (Biessels et al., 2014; Roberts et al., 2014). Insulin resistance is a key metabolic disturbance in prediabetes and T2DM. Its features of impaired insulin signaling and inflammation were also observed in AD patients, along with lower levels of insulin in cerebrospinal fluid and reduced activity of brain insulin-receptor (De Felice, 2013; Greene et al., 2015; Lin, 2008; Rani et al., 2016). It is reported that T2DM can promote higher level of β-amyloid protein (plaques) and hyperphosphorylation of Tau protein in cerebrospinal fluid, which resembles the pathological process of AD (Moran et al., 2015). Antidiabetic treatment can ameliorate the cognitive impairment of AD patients and improve their learning and executive processing function (Cai et al., 2018; Infante-Garcia et al., 2018). All those evidence indicate that hyperglycemia affects AD in a variety of forms.

Hippocampus and temporal lobe undergo atrophy in AD patients (Bernard et al., 2014; Gili et al., 2011). Moreover, many studies have indicated that T2DM patients also have brain atrophy (Li et al., 2016). However, the regions involved in different studies seem to be inconsistent and different from the typical AD biomarker-medial temporal atrophy (Bernard et al., 2014; Gili et al., 2011; Guo et al., 2014). In patients with T2DM or impaired fasting glucose (IFG), the volume of the brain was observed to be smaller, including the whole brain, cingulated cortex and temporal gyrus (Hou et al., 2016; Li et al., 2016). McIntyre et al. (2010) observed that individuals with T2DM exhibit volumetric abnormalities in both cortical and subcortical structures. The effect of prediabetes on brain volume is controversial. Some studies showed that prediabetes is associated with cognitive decline and lower brain gray matter volume (Markus et al., 2017), and even a higher plasma glucose level within the normal range is harmful to the brain (Cherbuin, Sachdev & Anstey, 2012). However, Schneider et al. (2017) found that the brain volume in the prediabetes population did not decrease significantly. In short, AD, IFG, and T2DM are all considered to be related to reduced brain volumes. Nevertheless, the association between hyperglycemia and brain volume changes in AD patients remains unclear. To the best of our knowledge, no studies have focused on the combined effects of AD and hyperglycemia (prediabetes or T2DM) on brain volume. In this study, to evaluate the associations between hyperglycemia and brain volume changes in AD patients, structural MRI was used to compare the brain volume among AD patients with different fasting glucose levels.

## Material and Methods

### Participants

AD patients were included in the study. The diagnosis of AD was confirmed according to the criteria of the National Institute of Neurological Disorders and Stroke - Alzheimer Disease and Related Disorders (Dubois et al., 2007) by neurologists from neurology department. Core diagnostic criteria were met and evidence of medial temporal lobe atrophy was acquired by MRI. Exclusion criteria were as follows: (1) history and clinical features of non - AD dementia; (2) severe cerebrovascular diseases, such as massive cerebral infarction or hemorrhage; head trauma or tumor; (3) cerebral structural abnormalities; (4) history of drug addiction, alcohol abuse, toxic or metabolic abnormalities; (5) histories of psychiatric diseases, such as schizophrenia or depression; (6) history of epilepsy or severe systemic illnesses; and (7) infectious diseases of central nervous system.

**Figure 1:**
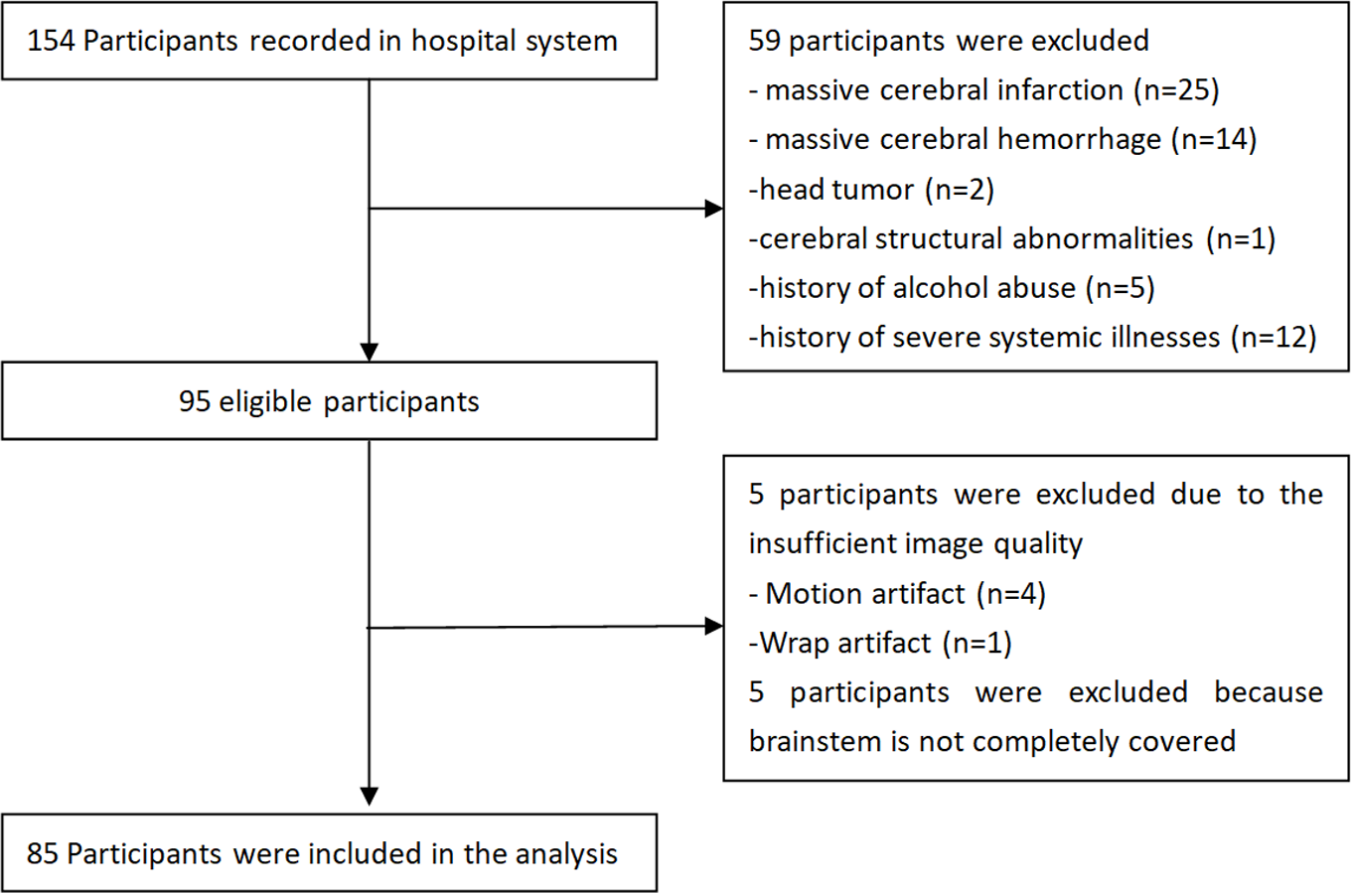
Flowchart of study participants.

Fifty-nine cases were excluded due to the exclusion criteria, five were excluded due to insufficient image quality, and five were excluded due to incomplete coverage of the brainstem during the magnetic resonance image (MRI) scan. Finally, 85 AD patients from outpatient and inpatient wards were included in the study (Fig. 1). These AD patients were divided into three groups based on the fasting glucose (FG) levels, as suggested by the American Diabetes Association (Association American Diabetes, 2010): (1) normal fasting glucose (NFG) group, FG levels <5.6 mmol/L (100 mg/dL); (2) impaired fasting glucose (IFG) group, FG levels of 5.6–6.9 mmol/L (100–125 mg/dL); and (3) T2DM group, FG levels ≥ 7.0 mmol/ L (126 mg/dL).

FG, serum total cholesterol, triglyceride, and blood pressure were obtained within 1 week prior to or posterior to the MRI examination. Cognitive performance was assessed using the Mini-Mental State Examination (MMSE). The study was conducted in accordance with the principles of the Declaration of Helsinki. The First Affiliated Hospital of Dalian Medical University has granted ethical approval to carry out the study within its facilities (Ethical Application Ref: YJ-KY-FB-2020-08). Informed consent for inclusion was waived due to the retrospective nature of the study.

### MRI data acquisition and analysis

MRI data acquisition was performed on a 3.0 T scanner (GE Signa HDxt) using axial T1WI, axial T2WI, axial T2 FLAIR, and sagittal three-dimensional (3D) T1-weighted gradient-recalled echo sequences. The parameters used for each sequence were as follows: (1) Sagittal 3D T1-weighted images: repetition time (TR) = 10.2 ms, echo time (TE) = 4.2 ms, inversion time (TI) = 450 ms, flip angle (FA) = 12°, FOV = 256 mm × 256 mm, Matrix = 256 × 256, thickness = 1.0 mm, gap = 0 mm, voxel size = 1.0 mm × 1.0 mm × 1.0 mm, totally 188 sagittal slices; (2) Axial T1WI: slice thickness = 6 mm, slice gap = 1 mm, TR = 2250 ms, TE = 24 ms, FOV = 240 mm × 240 mm, Matrix = 320 × 256, NEX = 1, Phase FOV = 0.9; (3) Axial T2WI: slice thickness = 6 mm, slice gap = 1 mm, TR = 5,000 ms, TE = mini, FOV = 240 mm × 240 mm, Matrix = 256 × 256, NEX = 2, Phase FOV = 0.8; (4) Axial T2 FLAIR: slice thickness = 6 mm, slice gap = 1 mm, TR = 9,000 ms, TE = 168 ms, FOV = 24 cm × 24 cm, Matrix = 256 × 192, NEX = 1.

Automatic segmentation and quantification of regional volumes and atrophy were performed using AccuBrain^®^ (BrainNow Research Institute, Shenzhen, Guangdong Province, China) (Abrigo et al., 2018; Yishan, 2017). Regions-of-interest-based segmentation methodology was adopted in AccuBrain^®^, which allows volume quantification of various anatomically defined brain structures; hence it could provide different types of information and could be more direct and intuitive when quantifying group differences. Multi-atlas-based segmentation method is used to automatically segment individual brain MRI. Preprocessing techniques including noise reduction, bias field correction, and intensity normalization were performed for image quality improvement. Technical aspects of brain segmentation and atrophy evaluation used have been previously described (Abrigo et al., 2018). In summary, brain parenchyma (i.e., gray matter and white matter tissue) and ventricular system were automatically segmented from 3D T1-weighted images by incorporating experienced radiologists’ prior knowledge, in which anatomical information can be transformed and applied to individual brain automatically. Based on the segmentation results, absolute volumes of 33 brain structures were computed. To correct for inter-subject head size variability in the analysis, relative volumes were calculated as the percentage of absolute volume by the total intracranial volume (ICV). Atrophy of cerebral lobes was computed as the ratio of cerebrospinal fluid volume to brain parenchymal volume (i.e., sum of white matter and gray matter volume) in the corresponding region (Yishan, 2017).

White matter hyperintensities (WMH) were automatically segmented from T2 FLAIR and T1-weighted images using an in-house developed pipeline previously published, which is a coarse-to-fine mathematical morphology method based on binary dilation, grayscale closing, binary reconstruction and grayscale reconstruction (Shi et al., 2013).

### Statistical analysis

Gender comparison among groups was performed using *χ*^2^ test. For other demographics, including physiological indices (serum total cholesterol, triglyceride, and blood pressure) and the cognitive scores, normally distributed variables were compared among three groups using one-way analysis of variance (ANOVA), while non-normally distributed variables were compared using non-parametric test. For regional volumetric measures, one-way ANOVA was used to assess the difference among three groups. Then the *p* values were corrected by applying the false discovery rate (FDR) correction. Further comparisons were performed using Tukey’s post hoc test to identify regions which two groups showed significant differences. One-way ANOVA was also used to assess WMH differences among the three groups. For regions with statistical significance, partial correlation analysis was used to evaluate their relationships with fasting glucose, controlling for age, education level, MMSE scores, cholesterol, triglyceride, and blood pressure. All the analyses were two-tailed, and *p* values <  0.05 were considered statistically significant.

## Results

Thirty-one male and 54 female AD patients were included with a mean age of 72.718 ± 7.867 years. According to the group division criteria, 45 patients were classified as AD_NFG group, 15 patients as AD_IFG group, and 25 patients as AD_T2DM group. The demographic and physiological indices (fasting glucose, serum total cholesterol, triglyceride, and blood pressure) and cognitive scores of the participants are presented in Table 1. No significant difference in age, gender, education level, and cognitive score were found among all three groups. Except for the fasting glucose level, no statistical difference was found in other physiological indices. All these ensure the comparability among the three groups.

**Table 1:** Demographics data of the study sample in three groups.

	AD_NFG	AD_IFG	AD_T2DM	*p*-value
Age, y	71.844(8.383)	74.267(9.316)	73.360(5.816)	0.527
Male, n(%)	16(0.356)	7(0.467)	8(0.320)	0.636
Education, y	9.556(4.467)	10.033(4.038)	10.060(4.447)	0.904
Fasting glucose, mmol/L	4.966(0.354)	5.860(0.207)	8.803(3.691)	**0.000**
Total cholesterol, mmol/L	5.169(1.116)	4.867(0.721)	5.138(0.963)	0.599
Triglyceride, mmol/L	1.157(0.485)	1.277(0.414)	1.174(0.336)	0.583
SBP, mm Hg	129.111(16.456)	137.333(17.512)	130.800(15.253)	0.203
DBP, mm Hg	80.333(7.863)	86.000(11.832)	80.600(7.948)	0.110
MMSE	14.111(7.334)	15.400(5.248)	14.800(7.858)	0.818

**Notes.**

NFG Normal fasting glucose IFG Impaired Fasting Glucose T2DM Type 2 Diabetes mellitus SBP systolic pressure DBP diastolic pressure y years

The data was presented as mean (SD) except gender.

**Figure 2:**
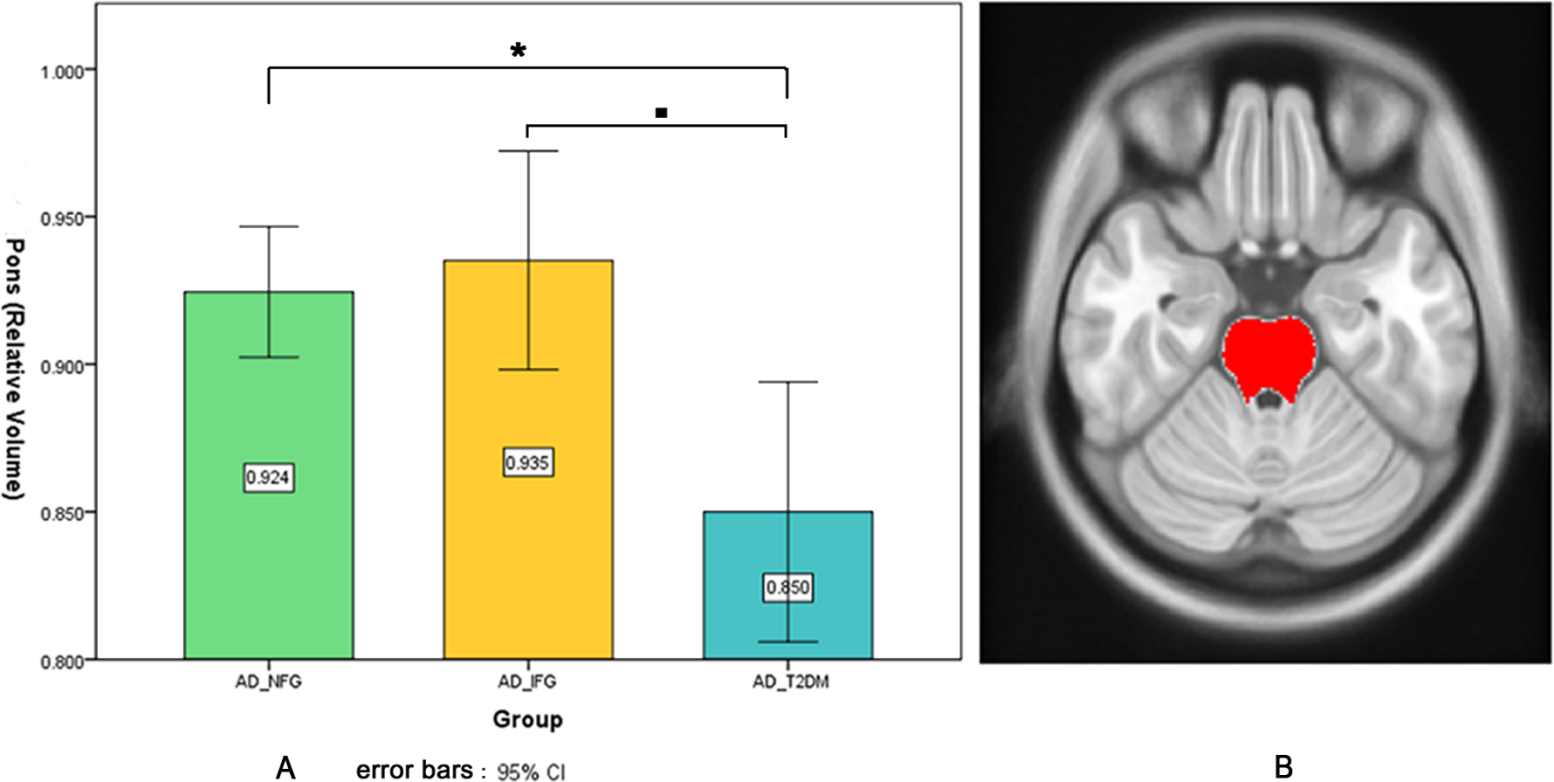
Pontine volumetric measures. (A) Pontine volumetric measures of AD_NFG, AD_IFG, and AD_T2DM groups. (B) The red area on the axial atlas represents Pons. * ■ FDR *p* < 0.05.

**Figure 3:**
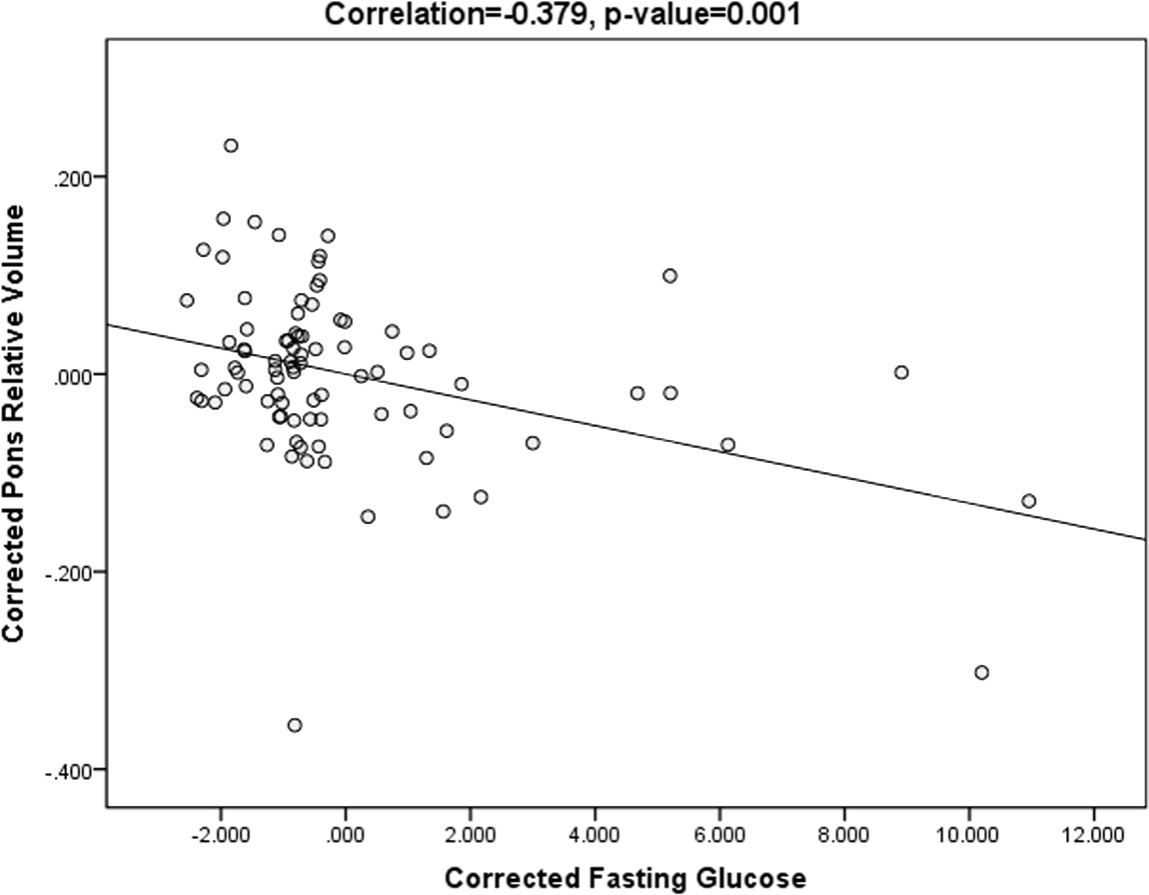
Scatter plots of pontine volume and fasting glucose level. Scatter plots showing the negative correlation between pontine volume and fasting glucose level, after correcting for age, education level, MMSE, cholesterol, triglyceride, and blood pressure (*r* =  − 0.379, *p* = 0.001).

Volumetric results showed no significant difference in ICV among the three groups. When examining regional volumetric measures, we found significant pontine volume difference among the three groups (uncorrected *p* = 0.001, FDR *p* = 0.033), but no significant difference in other regions. Post hoc test (Fig. 2) showed that the AD_T2DM group had significantly smaller pons (relative volume = 0.850 ± 0.107) compared with AD_IFG (relative volume = 0.935 ± 0.067, *p* = 0.003) and AD_NFG group (relative volume = 0.924 ± 0.074, *p* = 0.001). No significant difference was found between AD_NFG and AD_IFG group. Besides, fasting glucose level was found to have a significant negative correlation with pontine volume (*r* =  − 0.379, *p* = 0.001) after correcting for age, education level, MMSE, cholesterol, triglyceride, and blood pressure (Fig. 3). No significant difference was found in WMH among the three groups (uncorrected *p* = 0.683, FDR *p* = 0.855).

## Discussion

In this study, we examined the heterogeneity of brain regional volume in AD patients with different fasting glucose levels. We found that compared with the AD_IFG and AD_NFG groups, the pontine volume of AD_T2DM patients was smaller. In addition, correlation analysis revealed that fasting glucose level was negatively correlated with pontine volume. These results indicate that T2DM is associated with the brain volume changes in AD patients.

Previous studies have shown that AD, IFG, and T2DM are closely related (Degen et al., 2016; Greene et al., 2015; Kadohara, Sato & Kawakami, 2017; Lin, 2008; Moran et al., 2015; Rani et al., 2016; Zhang et al., 2017). Common pathological processes were found in these diseases. As a progressive neurodegenerative disease, AD is characterized by brain volume loss caused by neurodegeneration and brain cell destruction. Previous studies (Bernard et al., 2014; Matsuda, 2016; Schmidt-Wilcke et al., 2009) have shown that AD patients have more extensive atrophy compared to healthy population, involving both cortex and white matter, such as the hippocampus, corpus callosum, frontal and temporal lobes. Pontine atrophy has also been found in AD population (Mann, 1983; Lee et al., 2015), but it was less reported as compared with medial temporal lobe atrophy. A recent study showed that compared to the control group, bilateral volume loss in the pons was found in the mild AD group (Ji et al., 2020).

In this study, we found that the AD_T2DM group had a smaller pontine volume than the AD_IFG and AD_NFG groups. However, other structures (Matsuda, 2016; Schmidt-Wilcke et al., 2009) that typically show atrophy in the AD population did not demonstrate differences among the three groups. Hence, our findings suggest that for AD patients, their pons might be more susceptible to T2DM related atrophy than other structures. It might associated with the heterogeneous distribution of amyloid plaques (Braak & Braak, 1991) in AD brains. The distribution of amyloid plaques varied within cerebral regions. The pons is known to be relatively unaffected by amyloid deposition compared to other structures (Braak & Braak, 1991; Engler et al., 2006), and it is usually used as the reference structure for the study of cerebral glucose metabolism in AD (Cho et al., 2020; Grothe & Teipel, 2016). It is inferred that compared with structures mostly affected by amyloid deposition, pons could be more vulnerable to hyperglycemia in AD patients. Besides, T2DM is reported related to brainstem dysfunctions (Fuente-Martín et al., 2019), and significant associations were observed between metabolic syndrome (including hyperglycemia) and decreased gray matter volume of brainstem (including the pons) (Kotkowski et al., 2019). Previous studies have shown that FG is a risk predictor for T2DM with brainstem infarction (Ichikawa et al., 2012a; Ichikawa et al., 2010; Lu et al., 2011), and the brainstem is vulnerable to hyperglycemia (Ichikawa et al., 2012b). These evidence suggest the pons is related and vulnerable to hyperglycemia. Overall, our results indicate that among AD patients in different stages of T2DM development, cerebral structures generally presented similar atrophy patterns except for pons. Most cerebral structures, including hippocampus, temporal lobe and other regions previously shown to have atrophy in AD studies remained stable across groups. In the AD population, most regions may not shrink further as dysglycemia progresses, with the exception that the pons is a sensitive and key area associated with hyperglycemia. Pons of AD patients might shrink further as dysglycemia progresses from IFG to T2DM. The results also show that the brain atrophy rate could vary regionally, which is consistent with previous findings that brain pathological changes are regionally different and disease-stage specific (Byun et al., 2015; Tosun et al., 2010). In addition, compared with AD alone, patients with both T2DM and AD would show a distinctive pattern of brain atrophy, and T2DM may accelerate pontine atrophy in AD patients. Meanwhile, the pontine volume of the AD_NFG and AD_IFG groups was not significantly different in this study, which is consistent with the results of Schneider’s study (Schneider et al., 2017). In other words, mild hyperglycemia (IFG) did not cause pontine atrophy that is detectable by MRI, while T2DM is associated with significant pontine atrophy. This suggests that it is necessary to control the progression of hyperglycemia to avoid obvious pontine atrophy in AD patients.

Moreover, it was observed that the volume of pons was significantly negatively correlated with FG level, after correcting for MMSE scores, age, education level, cholesterol, triglyceride, and blood pressure. These results suggest that FG level is associated with pontine volume. This is consistent with existing studies which have shown significant association between hyperglycemia and brain atrophy (Li et al., 2016). Similar to our results, Cherbuin (Cherbuin, Sachdev & Anstey, 2012) showed that FG was positively correlated with hippocampal and amygdalar atrophy. Previous studies also observed that in the default mode network, FG level was positively correlated with the right middle temporal gyrus connection (Chen et al., 2015) and negatively correlated with fine motor skills (Zhang et al., 2018). Compared to non-diabetic participants, aged adults with T2DM showed lower CBF in predilection sites for AD pathology (Bangen et al., 2018). These provide evidence that FG level is related to both brain structure and function. Our results demonstrate that hyperglycemia is associated with a small pontine volume, suggesting that diabetes management is crucial for maintaining the pontine volume for AD patients. As it is known that the FG level of T2DM patients is reversible after medication, the follow-up question is whether the brain atrophy is also reversible.

In this study, there was no significant difference in WMH volume among the three groups. Thus, there was no evidence of a significant relationship between WMH volume and hyperglycemia, which might be attributed to the pathology of AD. WMH is usually deemed as an MRI sign of microvascular diseases. As a progressive neurodegenerative disease, the main pathological cerebral changes in AD are the deposition of *β*-amyloid protein and hyperphosphorylation of Tau protein. The typical MRI feature of AD is brain atrophy, and previous study has shown that AD is not necessarily associated with WMH volume (Sudre et al., 2017). Although T2DM presents small vessels and microvascular damage, the association between T2DM and WMH remains unclear (Brundel, Kappelle & Biessels, 2014). Some previous findings appear to be inconsistent with our result (Del Bene et al., 2015; Schneider et al., 2017), but De Bresser et al. (2018) also observed that there is no significant difference in WMH volume between T2DM and control group. More evidence is required to elucidate the relationship between WMH volume and hyperglycemia.

Our results have provided new insight into the association between hyperglycemia and brain volume changes in patients with AD. However, this study has several limitations that should be addressed in future research. First, longitudinal follow-up data was not included, so we were restrained from providing more in-depth results under the cross-sectional design. Second, the sample size was relatively small, particularly for the AD_IFG group, which limited the interpretation and generalizability of our results. However, as several studies have associated pontine degeneration with dysglycemia and AD (Ichikawa et al., 2012a; Ji et al., 2020; Lee et al., 2015), it may be possible to extend these findings to patients with comorbid dysglycemia and AD. However, further studies are needed on a larger cohort to confirm these preliminary findings. Third, the study sample only included the Chinese population and lacked ethnic diversity. Last but not least, because of missing data, the potential impact of other factors was not investigated in this study, such as disease duration and diabetes medication, which should be addressed in future research.

## Conclusions

AD patients with T2DM showed smaller pontine volume compared to those with normal blood glucose and IFG. Compared with AD alone, patients with both T2DM and AD would show a distinctive pattern of brain atrophy. T2DM may exacerbate pontine atrophy in AD patients, and FG level is associated with pontine volume. No evidence of a significant relationship between WMH volume and hyperglycemia was found.

##  Supplemental Information

10.7717/peerj.9801/supp-1 Supplemental Information 1 Raw data of demographic information of 85 participants (retrospective study) NFG= Normal fasting glucose, IFG= Impaired Fasting Glucose, T2DM= Type 2 Diabetes mellitus, SBP= systolic pressure, DBP= diastolic pressure

10.7717/peerj.9801/supp-2 Supplemental Information 2 Raw data of volume and WMH (retrospective study) Based on the segmentation results, atrophy of cerebral lobes was computed as its atrophic volume of cerebrospinal fluid, which was expressed as a percentage of total brain parenchymal volume. Volume from every other cerebral region was calculated as the percentage of its absolute volume by the total intracranial volume (ICV), where ICV was used to estimate the whole brain volume and correct the inter-subject head size variability.

10.7717/peerj.9801/supp-3 Supplemental Information 3 Results of regional volumetric measures (% of ICV) and WMH in the cohorts of AD

## Additional Information and Declarations

### Data Availability

The following information was supplied regarding data availability: The raw measurements are available in the Supplementary Files.
