# Supplementary material for: Association of impaired fasting glucose and Type 2 Diabetes Mellitus with brain volume changes in Alzheimer’s Disease patients analyzed by MRI: a retrospective study
Source: PeerJ. 2020 Aug 27;8:e9801. doi: 10.7717/peerj.9801 (PMC7456526; doi:10.7717/peerj.9801)
Supplement: Supplemental Information 3 [file peerj-08-9801-s003.docx]

Results of regional volumetric measures (% of ICV) and WMH in the cohorts of AD

|  | AD_NFG  mean(SD) | AD_IFG  mean(SD) | AD_T2DM  mean(SD) | p-value  (FDR) |
| --- | --- | --- | --- | --- |
| Midbrain | 0.373(0.022) | 0.373(0.015) | 0.362(0.023) | 0.567 |
| Pons | 0.924(0.074) | 0.935(0.067) | 0.850(0.107) | **0.033** |
| Medulla | 0.264(0.021) | 0.266(0.013) | 0.253(0.018) | 0.385 |
| SCP | 0.012(0.002) | 0.011(0.001) | 0.011(0.001) | 0.248 |
| Cerebellum | 9.213(0.670) | 9.149(0.447) | 8.834(0.714) | 0.515 |
| Hippocampus (L) | 0.203(0.023) | 0.204(0.038) | 0.208(0.032) | 0.930 |
| Hippocampus (R) | 0.216(0.027) | 0.213(0.036) | 0.223(0.033) | 0.844 |
| Amygdala (L) | 0.100(0.014) | 0.100(0.017) | 0.100(0.018) | 0.996 |
| Amygdala (R) | 0.119(0.018) | 0.115(0.019) | 0.118(0.024) | 0.930 |
| Ventral DC (L) | 0.132(0.012) | 0.132(0.009) | 0.129(0.009) | 0.844 |
| Ventral DC (R) | 0.145(0.012) | 0.144(0.009) | 0.141(0.012) | 0.813 |
| Thalamus-Proper (L) | 0.433(0.036) | 0.433(0.034) | 0.432(0.034) | 0.996 |
| Thalamus-Proper (R) | 0.405(0.035) | 0.399(0.039) | 0.400(0.033) | 0.930 |
| Caudate (L) | 0.205(0.023) | 0.206(0.021) | 0.209(0.030) | 0.930 |
| Caudate (R) | 0.232(0.025) | 0.225(0.019) | 0.235(0.038) | 0.899 |
| Putamen (L) | 0.324(0.036) | 0.327(0.034) | 0.325(0.038) | 0.996 |
| Putamen (R) | 0.310(0.031) | 0.306(0.033) | 0.310(0.035) | 0.996 |
| Pallidum (L) | 0.097(0.014) | 0.096(0.007) | 0.094(0.010) | 0.844 |
| Pallidum (R) | 0.093(0.012) | 0.090(0.010) | 0.089(0.010) | 0.827 |
| Accumbens-area (L) | 0.023(0.003) | 0.024(0.004) | 0.023(0.003) | 0.813 |
| Accumbens-area (R) | 0.026(0.004) | 0.025(0.004) | 0.024(0.004) | 0.770 |
| Frontal Lobe (L) - Atrophy | 44.153(6.866) | 45.393(8.563) | 45.404(10.440) | 0.930 |
| Frontal Lobe (R) - Atrophy | 41.184(6.425) | 41.720(7.645) | 43.852(10.647) | 0.827 |
| Occipital Lobe (L) - Atrophy | 20.527(5.394) | 20.553(2.472) | 19.160(5.038) | 0.844 |
| Occipital Lobe (R) - Atrophy | 16.198(4.774) | 16.567(3.690) | 15.591(4.186) | 0.930 |
| Temporal Lobe (L) - Atrophy | 31.107(5.730) | 32.113(6.350) | 34.080(14.068) | 0.827 |
| Temporal Lobe (R) - Atrophy | 23.909(6.850) | 23.753(5.196) | 26.520(7.595) | 0.770 |
| Parietal Lobe (L) - Atrophy | 49.504(11.933) | 49.873(11.327) | 45.276(10.051) | 0.770 |
| Parietal Lobe (R) - Atrophy | 43.796(12.141) | 42.153(10.891) | 41.952(10.097) | 0.930 |
| Cingulate Lobe (L) - Atrophy | 14.634(3.702) | 16.290(4.482) | 16.477(5.504) | 0.733 |
| Cingulate Lobe (R) - Atrophy | 22.280(5.301) | 24.820(6.559) | 24.996(8.856) | 0.733 |
| Insular (L) - Atrophy | 40.613(9.923) | 41.840(5.913) | 49.324(31.324) | 0.733 |
| Insular (R) - Atrophy | 23.329(7.104) | 22.473(6.361) | 27.644(11.134) | 0.515 |
| WMH | 0.950(0.804) | 1.057(0.811) | 1.135(0.981) | 0.855 |

Note: T2DM= Type 2 Diabetes mellitus, IFG= Impaired Fasting Glucose, NFG= Normal fasting glucose, L=left, R=right, DC=diencephalon, SCP=superior cerebral peduncle, WMH=white matter hyperintensities. *Atrophy is calculated as the brain atrophic volume of cerebrospinal fluid in the region (% of brain parenchymal volume).
